# Analyzing infection prevention and control program in Egyptian university hospitals: strengths and areas for improvement

**DOI:** 10.1186/s13756-026-01758-z

**Published:** 2026-05-18

**Authors:** Moustapha Ramadan, Nesrine Fathi Hanafi, Amany Mohamed Aboelenein, Asmaa Abdel-Aziz, Abeer Ezzat Elsayed, Mahmod Arabi, Hoda Mohamed Owais

**Affiliations:** 1 Community Medicine Department, Faculty of Medicine, Alexandria University, Alexandria, Egypt; 2 Department of Medical Microbiology and Immunology, Faculty of Medicine, Alexandria University, Alexandria, Egypt; 3 Department of Clinical Pathology, Faculty of Medicine, Tanta University, Tanta, Egypt; 4 Department of Clinical Pathology, Faculty of Medicine, Assiut University, Assiut, Egypt; 5 Department of Medical Microbiology and Immunology, Faculty of Medicine, Suez Canal University, Ismailia, Egypt; 6 Suez Canal University, Ismailia, Egypt

**Keywords:** IPCAF, Core components, Patient safety, Compliance, National

## Abstract

**Background:**

Healthcare-associated infections are a major global health problem. Published international guidelines aim to provide technical support for countries and healthcare facilities to implement efficient infection prevention and control (IPC) programs and evaluate them.

**Aim:**

This study aims to evaluate Egyptian university hospitals’ current IPC program and activities status, using an international standardized tool. Afterwards, the identification of existing strengths and weaknesses of the IPC program is needed for improvement.

**Method:**

A cross-sectional study design using a convenience sample to assess the implementation of the IPC program at 26 healthcare facilities running the IPC activities for at least 5 years, amounting to > 11,000 beds, from four selected Egyptian Universities. The WHO IPCAF assessment tool and scoring were used to assess the level of compliance for each core component.

**Results:**

The weighted median of participating facilities was 600 ( IQR = 571.25–646.88). The arithmetic mean of participating facilities was 587.19. None of the studied facilities had an inadequate level of IPC, and (50%) of facilities showed an advanced level. IPC guidelines had the highest mean score (83.46%), followed by the IPC program (76.73%). On the other hand, the multimodal strategies in IPC had the lowest mean score (65.57%) following the IPC training and education (69.23%).

**Conclusion:**

Although the IPC program is appropriately implemented in the studied hospitals, some challenges exist, and enhancement of multidisciplinary integration optimizes multimodal IPC strategies, implementation of antimicrobial stewardship, training programs, and monitoring and feedback systems are required.

**Supplementary Information:**

The online version contains supplementary material available at 10.1186/s13756-026-01758-z.

## Introduction

Healthcare-associated infections (HAIs) are a major global health problem. They not only lead to increased illness, prolonged hospitalization, increased resistance to antimicrobials, reduced quality of life with long-term disabilities, but also place a heavy financial burden on healthcare systems and result in death [1]. World Health Organization (WHO) has published *“Guidelines on Core Components of Infection Prevention and Control Programmes at the National and Acute Healthcare Facility Level*” followed by “*Minimum Requirements for Infection Prevention and control programs*”. These guidelines aimed by setting 8 core components in infection prevention and control (IPC) to provide evidence-based recommendations on the IPC programs that are required to be in place at the national and acute facility level to prevent HAIs, to combat antimicrobial resistance (AMR); and to support countries and healthcare facilities to develop or strengthen IPC programs and strategies through the provision of evidence- and consensus-based guidance that can be adapted to the local context. They targeted not only IPC focal points but also policymakers, senior managers, stakeholders, and other professionals with a mandate or interest in developing or strengthening IPC programs at national, subnational, and facility levels [2, 3]. 

In Egypt, earlier in this century, efforts were undertaken by the Ministry of Health and Population in collaboration with the US Naval Medical Research Unit No. 3 and the WHO to develop a national organizational structure, IPC guidelines, and a comprehensive IPC training program [4]. The first national IPC guidelines were published in 2003, followed by 3 updates; the latest version was in 2020 [5]. This was followed by implementing a surveillance program for HAIs and applying the multimodal strategies for IPC to protect patients, healthcare workers, and the environment from the hazards of infection transmission [6, 7]. 

The WHO Infection Prevention and Control Assessment Framework (IPCAF) is a systematic tool for evaluating and improving IPC programs in healthcare facilities. It helps assess the current state of IPC activities, identify strengths and weaknesses, and track progress over time. The IPCAF is primarily intended for self-assessment by healthcare facilities, but can also be used in joint assessments with external stakeholders [8]. The IPCAF assessment tools have many benefits by identifying areas for improvement; the IPCAF can help facilities enhance their IPC practices. Stronger IPC practices reduce the risk of HAI. Improved IPC contributes to better quality of patient care. The IPCAF provides a structured way to assess and improve IPC, making it a more evidence-based approach [9–12]. 

The IPCAF assessment tool is characterized by a closed-form questionnaire with a scoring system. The framework is structured around eight core components of IPC. It is self-administered and aligned with WHO guidelines on core components of IPC programs [8]. 

WHO conducted a global survey during 2019 to assess IPC in healthcare facilities over the 6 regions, and the Eastern Mediterranean region represented 11.8% of the total responses, with no clarification for each country’s data [11]. A previous study was conducted in Egypt to assess the IPC measures at 3 primary healthcare facilities using different national and international tools other than IPCAF [13]. Therefore, studying the IPC measures more comprehensively, wider scope, and using a standardized international tool makes this study of utmost value.

This study aims to evaluate Egyptian university hospitals’ current IPC program and activities status, using a standardized tool. Afterwards, the identification of existing strengths and weaknesses of the IPC program that needed for improvement.

## Subjects and methods

### Design

A cross-sectional study using convenience sampling from four Egyptian universities was conducted in July and August 2025.

### Study setting

The four elected Egyptian Universities are composed of 31 university healthcare facilities providing primary, secondary, and tertiary care with 11,343 beds. These centers include, in addition to outpatient and daycare services, the intensive care units, the following non-ICU departments: surgical, medical, orthopedics, cardiology, cardiothoracic, otolaryngology, ophthalmology, oncology, pediatric oncology, obstetrics and gynecology, pediatric surgery, neonatal, organ transplant, nephrology, burn, and neuropsychiatry units.

### Selection criteria

Convenient sample of 4 Egyptian universities covering different geographical areas in Egypt (Upper Egypt, Delta, North west, and East regions). Eligible criteria from the selected universities were acute secondary or tertiary care centers with at least 5 years of IPC activities implementation. The Selection for 5 years of IPC implementation was based on two rationales: the first is that the healthcare facilities will have enough to implement the required IPC core components, overcome any challenges, and build channels of trust with the hospital administration and stakeholders. The second and most important is the resilience and recovery from the COVID-19 pandemic. With the exclusion of daycare, outpatient clinics, long-term care and rehabilitation facilities, and facilities with less than 5 years of IPC activities implemented, 5 were excluded from the study, with 26 healthcare facilities included in the study and 10,215 beds.

### Study tools

We used the WHO-IPCAF questionnaire to assess the core components of an IPC program in the selected health facilities. The IPCAF is a structured, closed-form questionnaire with an associated scoring system. This framework is designed to assess the current IPC situation at the health facility, specifically the existing IPC activities/resources, and identify strengths and gaps that can inform the development of plans. It is a diagnostic tool for health facilities to identify relevant problems or shortcomings that require improvement and pinpoint areas where they can meet international standards and requirements. Moreover, it can serve as a tool for ongoing evaluations to document progress over time and facilitate improvement. This tool is structured according to the recommendations in the WHO Guidelines on core components (CC) of IPC programs at the acute healthcare facility level. It is divided into eight sections, reflecting the eight WHO IPC core components, which are then addressed by a total of 81 indicators. These indicators are based on evidence and expert consensus and have been framed as questions with defined answers to provide an orientation for assessment.

The list of core components is as follows:

- CC1-IPC Program
- CC2-IPC Guidelines
- CC3-IPC Training and Education
- CC4-HAIs Surveillance System
- CC5-Multimodal Strategies in IPC
- CC6-Monitoring/Audit/Feedback
- CC7-Workload, Staffing, and Bed Occupancy within Facility
- CC8-Environments, Materials, and Equipment in the Facility

Each component (CC) in the tool has an equal score, i.e., 100 per component. The maximum cumulative score of all eight components is 800. For each section, questions are formatted as “Yes/No”, single or multiple choices, with a numerical score assigned to each response depending on how crucial the question is for IPC standards.

For strengths and weaknesses analysis, each core component was analyzed separately based on the number of common positive attributes among the participating healthcare facilities. The more common positive answers, the more considered as a point of strength for the core component under study. Likewise, the more negative common answers, the more considered as a weakness for the core component.

Based on the overall score achieved in the eight sections (on a total of 800), the facility is assigned to one of four progress levels of IPC implementation, thus:

- Inadequate (0–200): IPC core components implementation is deficient. Significant improvement is required.
- Basic (201–400): Some aspects of the IPC core components are in place, but not sufficiently implemented. Further improvement is required.
- Intermediate (401–600): Most aspects of the IPC core components are appropriately implemented. The facility should continue to improve the scope and quality of implementation and focus on the development of long-term plans to sustain and further promote the existing IPC program activities.
- Advanced (601–800): The IPC core components are fully implemented according to the WHO recommendations and appropriate to the needs of the facility.

### Data collection

Before the study data were collected, online meetings were held with the IPC unit/team head of the selected facilities to go over the study’s design and the introduction of the questionnaire form to ensure the accuracy and consistency of results. In order to ensure data confidentiality, a special code was created for each institution in the study. Data collection was made by members of the IPC team of the selected facility. Data collected was verified at the first level by the IPC unit/team head, then by the principal investigator to ensure uniformity of collected data.

### Statistical analysis

The data collected was entered into a Microsoft Excel sheet, cleaned, and exported to the Statistical Package for Social Sciences (SPSS version 27.0, IBM, Armonk, NY, USA) statistical software for analysis. Descriptive statistics were done to determine the IPC status of the various health facilities. The Kruskal-Wallis H Test was used to identify statistical differences in the core components between the four universities, and Bonferroni corrected post hoc analysis was performed.

### Ethical considerations

The study was approved by the Research Ethics Committee of Alexandria University Medical School IRB number:00012098.

## Results

**Fig. 1:**
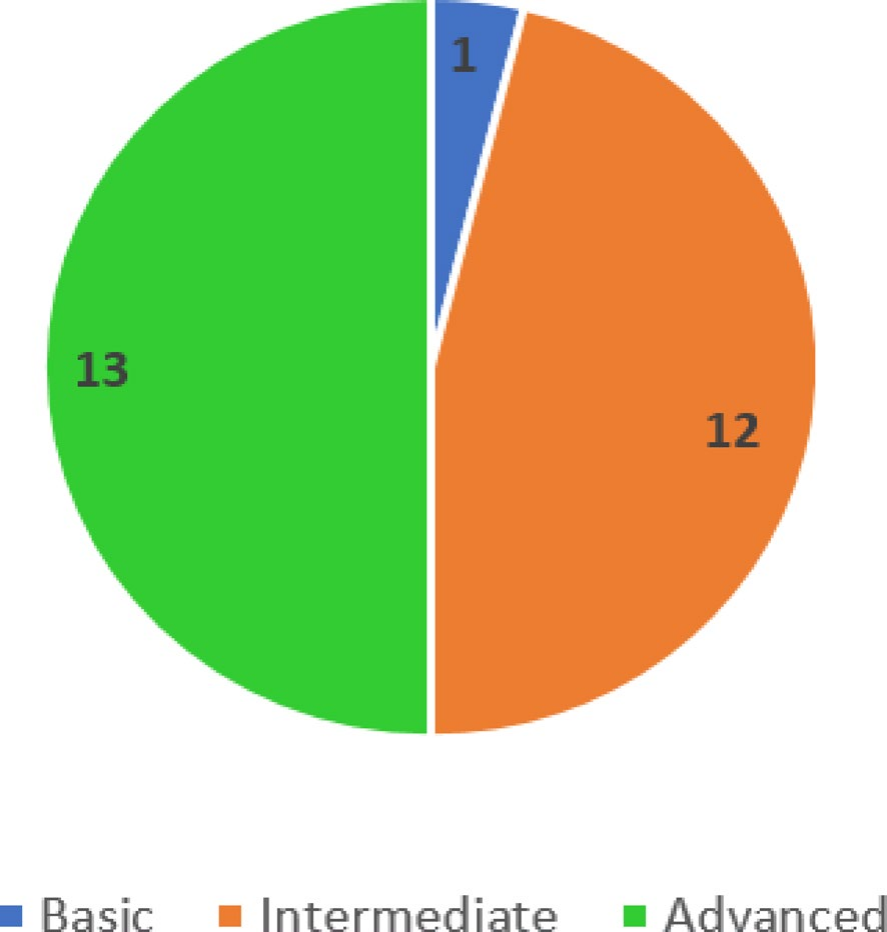
Distribution of healthcare facilities according to their IPC level in four studied universities in Egypt, August 2025

**Fig. 2:**
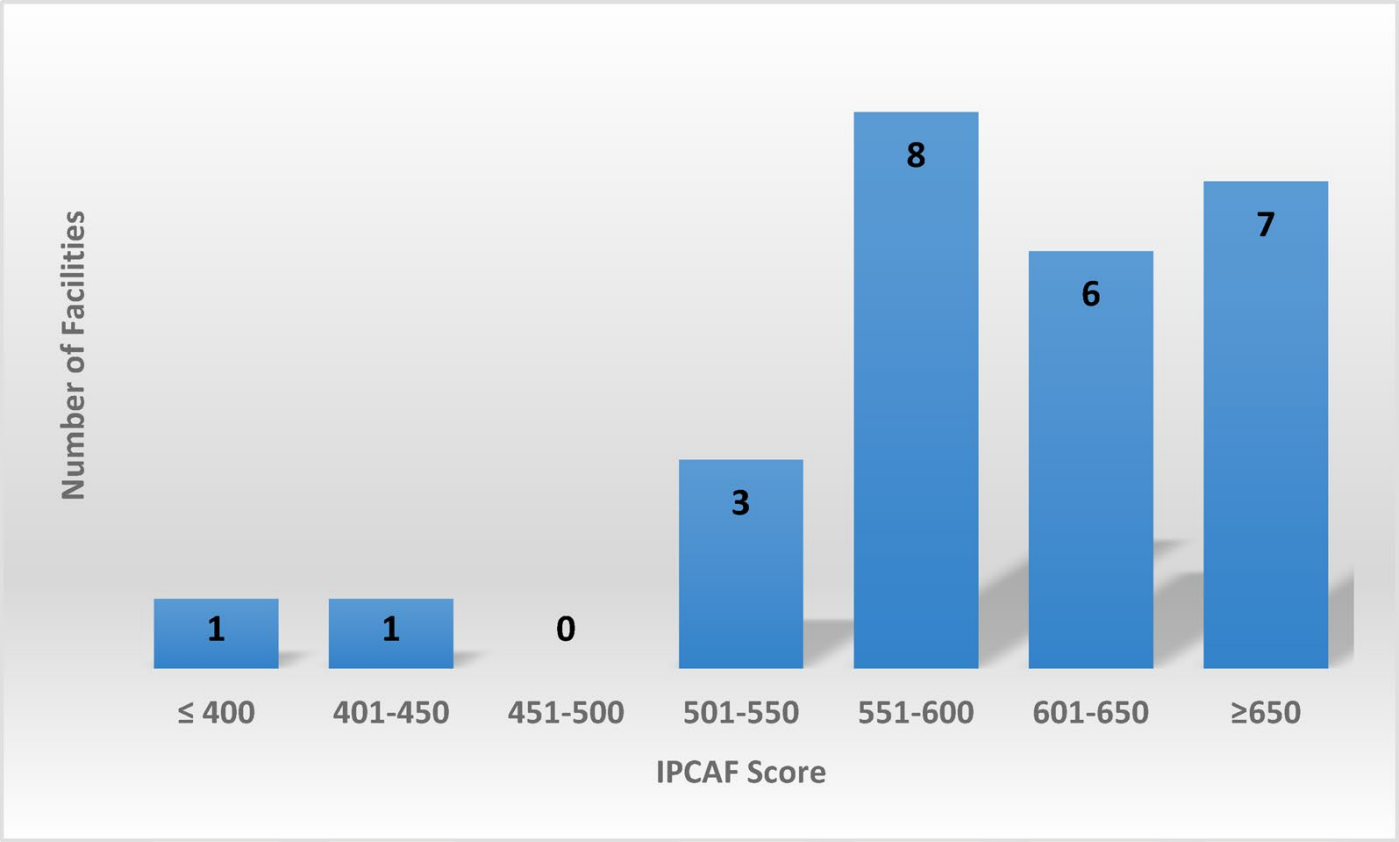
Distribution of healthcare facilities according to their IPCAF score in four studied universities in Egypt, August 2025

From the 26 participating university healthcare facilities, none showed an inadequate level of IPC, 1 basic, and (50%) of facilities showed an advanced level. (Fig. 1) Concerning the scoring level, no facility had scored more than 700 points, 1 facility had less than 400 points, and the highest range (30%) of the facilities was between 550 and 600 points. (Fig. 2)

**Fig. 3:**
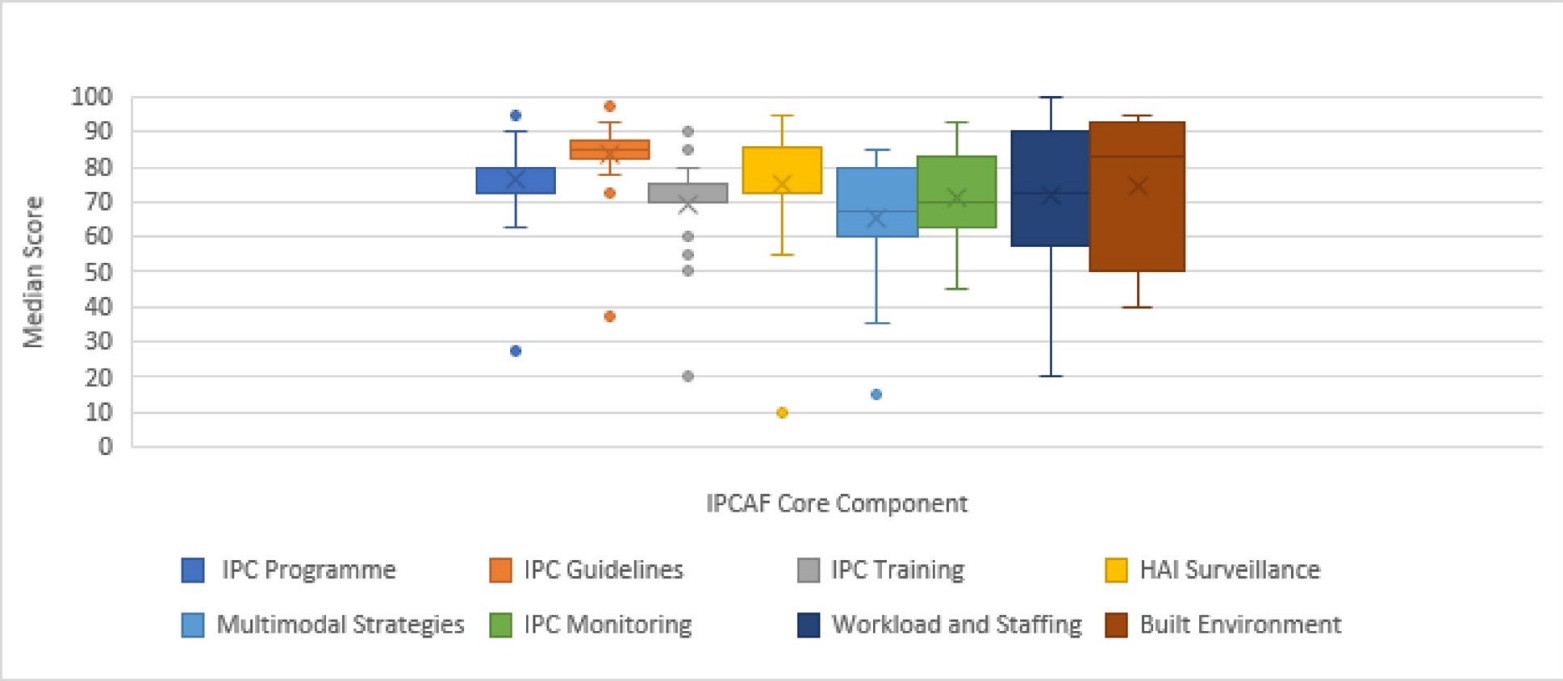
Distribution of IPCAF core components score for healthcare facilities in four studied universities in Egypt, August 2025

The detailed analysis of the 8 core components per healthcare facility showed that the lowest marked score was 10 in the surveillance component (CC4), and this was in 1 facility. This facility marked the lowest score in 7 core components out of 8. The highest marked score was 100 in core component 7 and was achieved by 5 facilities. The same 5 facilities marked the highest score of 95 for the IPC built environment (CC8). Pooling the results of the studied healthcare facilities, the IPC guidelines (CC2) had the highest scores, followed by the IPC program (CC1). The multimodal strategies in IPC (CC5) had the lowest score following the IPC training and education (CC3). Figure 3 and Table S1 provide more details on each hospital’s bed capacity and clinical specialization.

Grouping the participating 26 healthcare facilities under the 4 universities displayed that 2 universities had an intermediate IPC level, and 2 universities had an advanced IPC level. The mean of each core component at the 4 universities is presented in Table 1. The weighted median of participating facilities was 600 (IQR = 571.25-646.88). Table S2 The Kruskal-Wallis H Test indicated that there was a statistically significant difference between the IPCAF scores in the 4 universities, *H* (3) = 13.38, *p =* 0.004. The corrected α using the Bonferroni correction method is 0.008333 Table 2.

Comprehensive analysis of the core components elements, identifying the strengths and areas of improvement for each component, is highlighted and summarized in Table 3.

**Table 1:** Mean scores of core components per university healthcare facilities in four studied universities in Egypt, August 2025

Core component	Suez canal university *n* = 1	Assiut university *n* = 6	Alexandria university *n* = 8	Tanta university *n* = 11
C1-IPC program	62.5 (N/A)	72 (± 23.04)	77 (± 11.03)	80 (± 0)
C2-IPC guidelines	77.5 (N/A)	75.5 (± 19.71)	88.5 (± 5.37)	85 (± 0)
C3-IPC training and education	50 (N/A)	61.5 (± 22.95)	77 (± 14.39)	70 (± 0)
C4-HAIs surveillance system	55 (N/A)	74 (± 31.72)	83 (± 9.97)	73 (± 0)
C5-multimodal strategies in IPC	55 (N/A)	61 (± 24.37)	66.5 (± 16.51)	68 (± 10.29)
C6-monitoring/audit/feedback	45 (N/A)	79(± 16.71)	78.5 (± 12.4)	66 (± 3.86)
C7-workload, staffing, and bed occupancy within facility	50 (N/A)	55 (± 20)	67 (± 11.13)	84 (± 18.32)
C8-environments, materials, and equipment in the facility	46 (N/A)	49 (± 4.4)	67.5 (± 15.43)	94 (± 1.28)
Total	441	527	605	619

**Table 2:** Post hoc analysis of core components of the studied healthcare facilities in four universities in Egypt, August 2025

University	Assiut	Alexandria	Tanta
Suez	−6.63	−13.75*	−15.13**
Assiut	0	−7.13	−8.5
Alexandria	−7.13	0	−1.35

* *p =* 0.003

** *p =* 0.001

**Table 3:** Strengths and areas of improvement per core components among healthcare facilities in four studied universities in Egypt, August 2025

Core component	Strength	Areas for improvement
C1-IPC program	IPC program exists with clearly defined objectives and an annual action plan. A devoted full-time IPC team or focal person is present at the facility. A senior clinical staff member is represented at the IPC committee.	Dedicated budget for IPC program and activities
C2-IPC guidelines	The available guidelines at facilities are consistent with national and international guidelines. The IPC team monitors regularly the implementation of the guidelines	Guidelines for the prevention of multidrug-resistant organisms spread should be implemented. Tailored antimicrobial stewardship guidelines must be developed.
C3-IPC training and education	An IPC expert leads the training program at the facilities. Newcomers’ employees receive an IPC training/ orientation lecture. There is ongoing development and or continuous education for IPC staff.	Patients and family members should be targeted for IPC health education. A tailored IPC training/ education for administrative and managerial staff should be created. Existing staff must have refreshing training at least once annually.
C4-HAIs surveillance system	Clear, robust organization for surveillance activities with standardized methods of data collection and case definitions. The surveillance activities are in line with facility needs and priorities.	Surveillance should include analysis of multidrug-resistant organisms regularly. Data generated from surveillance should be shared with frontliners and non-clinical managers regularly.
C5-multimodal strategies in IPC	Monitoring compliance with process and outcome indicators for IPC activities and interventions. Reminders, posters, and other awareness-raising tools to promote the interventions are available and clearly displayed.	A multidisciplinary team is essential for the efficient implementation of IPC multimodal strategies. Linkage with other consistent clinical departments, such as Microbiology, Clinical Pharmacy, healthcare quality, and patient safety, is important to develop and promote IPC multimodal strategies.
C6-monitoring/audit/feedback	Trained personnel responsible for monitoring/audit of IPC practices, and feedback is present at the facilities. Monitoring hand hygiene compliance using the WHO tools and cleaning the ward environments are implemented in all the facilities.	Monitoring and feedback of IPC processes and indicators should be aimed at improvement and behavioral change. Assessing safety cultural factors using different tools should be planned.
C7-workload, staffing, and bed occupancy within facility	There is a system in place at participating facilities to address staffing levels that are deemed to be too low. It is uncommon to find beds with patients standing outside the patient rooms, even in emergency departments.	Staffing loads are not consistent with workload, and standard staffing needs should be properly assessed using standard indicators/tools. The design of wards, including the emergency department, in most of the participating facilities is not in accordance with international standards regarding bed capacity.
C8-environments, materials, and equipment in the facility	Water services are available at all times and of sufficient quantity for all uses. PPE is available at all times and in sufficient quantity for all uses for all healthcare workers. There is an approved treatment technology for infectious and sharp waste, and none of the facilities use an incinerator.	Records of cleaning should be completed, signed, and updated. The facilities should provide more single-patient rooms for treating communicable diseases and airborne transmitted infections. A wastewater system should be implemented and functioning reliably.

## Discussion

This study enables us to assess the IPC Process and framework in four Egyptian university hospitals representing four geographical areas in Egypt (Upper Egypt, Delta, North west, and East regions).

Among the 26 healthcare facilities studied, 13 (50%) achieved an advanced level of IPCAF scoring, and 7 had a score of more than 650. Only one healthcare facility had a basic level. The weighted median of participating facilities was 600 ( IQR = 571.25–646.88), higher than that of lower-middle-income countries (LMICs), 500.4 (IQR = 450.5–705), but less than that of participating facilities from the Eastern Mediterranean region, 715 (IQR = 632.5–740) in the WHO global survey [11]. Furthermore, this score variation, ranging from basic to advanced levels, was also observed in other LMICs like India (median = 620), Indonesia ( median = 620), and Pakistan (median = 405), indicating variable IPC practices among different facilities [14–17]. However, this was still better than findings from Syria (median = 167.5), where no facilities achieved the intermediate or advanced levels [18]. While a national survey on Turkey showed that (73.5%) of facilities had an advanced level, with a median of 668 [19]. On the other hand, these findings were in contrast with studies from high-income countries (HIC) [20, 21], which demonstrated more advanced levels in IPCAF scores [11, 17]. 

The infection control guidelines (CC2) and infection control programme (CC1) were the highest score among all 8 components, with mean scores (83.46%) and (76.7%) respectively. This indicated the commitment of Egyptian healthcare facilities to support, implement, and promote the infection control policies and practices. This was in accordance with the WHO global survey and single hospital-based Libyan study, which showed that CC1 and CC2 represented the highest score (95%) and (97.5%), respectively [11, 22]. On the other side, the lowest score in all core components was Multimodal Strategies in IPC (CC5), indicating that the understanding and application of multimodal strategies were insufficiently developed, and highlights the importance of implementing multiple infection control practices in an integrated way. Surprisingly, comparable findings were detected in both lower-middle-income countries from the global survey and by Tahir et al. [11, 16] and the HICs Austria and Germany [20, 23]. 

The presence of effective multiple national programmes supporting infection control practices with a dedicated team was a key strength in CC1, and the need for a dedicated budget was the main area of improvement. In contrast, other studies reported that (78%) and (78.7%) of hospital studies had a dedicated budget [22, 24]. So hospital management should strategically allocate resources to ensure the effective implementation of infection control practices.

Despite the availability of national and international guidelines, effective antimicrobial stewardship remains inconsistent. Successful programs necessitate stringent national-level oversight to standardize prescribing practices and provide evidence-based justifications for empirical antibiotic selection. By aligning policy with clinical evidence, healthcare systems can secure strategic advantages, such as preserving ‘last-resort’ antibiotics, mitigating the evolutionary pressure toward multidrug-resistant organisms (MDROs), and improving patient outcomes. Addressing these challenges requires extensive multidisciplinary collaboration for effective implementation and monitoring. Strengthening these initiatives would allow institutional performance—specifically within the WHO IPCAF Core Components—to reach the benchmarks established in high-income settings, such as German hospitals [23]. 

As a fundamental pillar for interrupting transmission pathways and reducing healthcare-associated infections (HAIs), IPC education and training (Core Component 3) must be systematically prioritized [25]. Evidence from both high-income and LMIC validates the necessity of an organized educational framework. Such programs should extend beyond frontline clinicians to include tailored initiatives for administrative leadership, patient-family awareness campaigns, and periodic refresher modules to ensure sustained competency across the healthcare workforce [26–28]. 

The healthcare-associated infections surveillance system was the third highest core component in our assessment, with a score of 75/100. This was due to the previously established HAI surveillance system at university hospitals, as well as to the national initiative to implement an effective electronic surveillance system. This may account for the observed discrepancy with other LMICs, which had no or a poor HAI surveillance system [11, 16, 18, 22]. 

Although well-trained IPC personnel were available for monitoring/audit of IPC practices, a strong focus is still needed for monitoring the consumption/usage of antimicrobial agents and multidrug-resistant organisms. This was in accordance with the results shown in the Austrian assessment that revealed a high proportion of hospitals didn’t conduct an antimicrobial drug-resistant surveillance [20]. Healthcare facilities need to raise awareness about the risks of antibiotic-resistant organisms and the importance of tracking antibiotic consumption. Feedback is a crucial part of IPC that improves patient safety and reduces HAIs. It encourages behavioural changes, ensuring staff regularly follow procedures like hand hygiene. The use of more effective feedback methods needs to be improved in all healthcare facilities. Several other studies from African countries revealed that the lowest score was in IPC monitoring and evaluation [1, 9, 29]. 

While the mean score for Core Component 7 was (71.5%), the staffing levels still fall short of the standard needed to manage the patient workload effectively. The design of wards, including the emergency department, in most of the participating facilities was not in accordance with international standards regarding bed capacity, but it was uncommon to find beds with patients standing outside the patient rooms, even in emergency departments. This was in accordance with the results of a national survey on German hospitals [23]. The inadequate staffing requires strong support and careful planning from high-level management. Policymakers must focus on ensuring a standard patient-to-staff ratio and making sure ward designs meet international standards for bed capacity.

For Core Component 8 (CC8), the facilities demonstrated clear strengths in a few areas. They had a continuous and ample supply of water, and personal protective equipment was consistently available in sufficient quantities for all healthcare staff. All facilities also used an approved method for treating infectious and sharp waste, avoiding the use of incinerators. Yet, several limitations were also identified. Cleaning records were not consistently completed, signed, or updated. There is a need for more isolation rooms to effectively treat patients with infectious and airborne diseases. Additionally, a reliable wastewater system has yet to be implemented. The CC8 represents the infrastructure of the healthcare facilities, which depends on the financial level of the country, so the higher score achieved by HICs like Japan [21]. and lower scores observed in LMICs [9, 17, 30].

## Limitations

Since the IPCAF is a self-reported tool, the responses may have been influenced by social desirability bias. This means participants might have chosen answers that they felt were more favourable or socially acceptable rather than providing completely truthful responses. Additionally, the study’s findings are limited by its relatively small sample size, and do not represent some vital areas like the capital or nearby cities. A final limitation is selecting healthcare facilities with 5 years of IPC implementation; this would be biased towards higher-performing programmes in Egypt.

## Conclusion

The studied Egyptian university hospitals have a solid foundation for infection prevention and control (IPC), with policies and resources largely in place. However, the study shows that there are significant challenges in the real-world application of these measures. Key areas needing improvement include securing dedicated funding, strengthening antimicrobial stewardship, enhancing training, and implementing more effective monitoring and feedback systems. Closing these gaps is crucial for improving patient safety and reaching the high standards of IPC performance seen in HICs.

## Supplementary Information

Below is the link to the electronic supplementary material.

Supplementary Material 1

## Data Availability

The datasets used and/or analysed during the current study are available from the corresponding author on reasonable request.

## Abbreviations

AMR: Antimicrobial resistance
CC: Core components
HAI: Healthcare-associated infection
IPC: Infection prevention and control
IPCAF: Infection prevention and control assessment framework
ICU: Intensive care unit
LMICs: low and middle-income countries
MDRO: Multidrug-resistant organisms
WHO: World Health Organization
