# Supplementary material for: Analyzing infection prevention and control program in Egyptian university hospitals: strengths and areas for improvement
Source: Antimicrob Resist Infect Control. 2026 May 18;15:72. doi: 10.1186/s13756-026-01758-z (PMC13185206; doi:10.1186/s13756-026-01758-z)
Supplement: Supplementary file 1 — Supplementary Material 1 [file 13756_2026_1758_MOESM1_ESM.docx]

**Table S1: Specialization and bed capacity for healthcare facilities in four studied universities Egypt, August 2025**

| **University Name** | **Specialization** | **Bed Capacity** |
| --- | --- | --- |
| Suez Canal | General | 381 |
| Assiut | Cardiology and Cardiothoracic | 380 |
|  | Pediatric | 620 |
|  | Hepatology | 240 |
|  | Neurology and Neurosurgery | 430 |
|  | Internal Medicine and Neonatology | 40 |
|  | General | 1400 |
| Alexandria | Pediatric Oncology and surgery | 78 |
|  | Orthopedic and Neuropsychiatry | 412 |
|  | Pediatric and Pediatric Surgery | 315 |
|  | Obstetrics, gynecology and neonatology | 359 |
|  | General surgical and transplant | 260 |
|  | Pediatric | 332 |
|  | Surgical | 275 |
|  | General | 1950 |
| Tanta | Oncology | 96 |
|  | Pediatric Oncology | 66 |
|  | Urology | 180 |
|  | Pulmonology and chest diseases | 115 |
|  | Ophthalmology | 159 |
|  | Pediatric | 214 |
|  | Emergency and Surgical | 265 |
|  | Medical | 128 |
|  | Medical – Neuropsychiatry | 173 |
|  | General | 516 |
|  | Surgical | 831 |

**Table S2: Descriptive Statistics of core components scores for healthcare facilities in four studied universities Egypt, August 2025**

| **Max** | **Min** | **Median (IQR)** | **Mean (STD)** | **Core Component** |
| --- | --- | --- | --- | --- |
| 95 | 27.5 | 80 (6.875) | 76.73 (± 12.4) | **C1- IPC Program** |
| 97.5 | 37.5 | 85 (3.75) | 83.46 (± 10.49) | **C2- IPC Guidelines** |
| 90 | 20 | 70 (5) | 69.23 (± 14.19) | **C3- IPC Training and Education** |
| 95 | 10 | 72.5 (11.875) | 75.09 (±16.22) | **C4- HAIs Surveillance System** |
| 85 | 15 | 67.5 (20) | 65.57 (±15.64) | **C5- Multimodal Strategies in IPC** |
| 92.5 | 45 | 70 (20) | 71.34 (± 13.06) | **C6- Monitoring/Audit/Feedback** |
| 100 | 20 | 72.5 (26.25) | 71.54 (±20.58) | **C7-** **Workload, Staffing, and Bed Occupancy within Facility** |
| 95 | 40 | 83 (41.875) | 74.21 (±21.06) | **C8- Environments, Materials, and Equipment in the Facility.** |
